# Retraction: Identification of immune-related lncRNAs to improve the prognosis prediction for patients with papillary thyroid cancer

**DOI:** 10.1042/BSR-2020-4086_RET

**Published:** 2024-03-13

**Keywords:** ceRNA networkp, immune-related lncRNA, papillary thyroid cancer, PD1

This article is being retracted from Bioscience Reports at the request of the Editor-in-Chief and the Editorial Board following receipt of a notification from a reader, alerting the Editorial Board to multiple images within the article that seem to appear in other papers by different authors. Specifically:

- Figure 6E NC panel which appears as Figure 3B miR-1236-3p mimics SW620 panel from Zhao et al 2021 (DOI: 10.3389/fonc.2021.688882), Figure 2B MDA-MB-231 200 panel from Wang et al 2020 (DOI: 10.1038/s41419-020-2690-y), Figure 10H shCD200R1 panel from Ling et al 2021 (DOI: 10.3389/fmolb.2020.603701), Figure 6E MG63 shTLR7 panel from Cao et al 2020 (DOI: 10.2147/OTT.S256617) [Retracted], and Figure 2E sh-KIF4A panel from Feng et al 2020 (DOI: 10.1186/s12957-020-02088-z)
- Figure 6E Si-SLC26A4-AS1 panel which appears as Figure 2B MDA-MB-231 400 panel from Wang et al 2020 (DOI: 10.1038/s41419-020-2690-y, and Figure 6E MG63 WT panel from Cao et al 2020 (DOI: 10.2147/OTT.S256617) [Retracted]

The authors have been contacted with regards to the retraction and have not responded to the Journal's queries or the concerns raised. Given the extent of the issues raised, the Editorial Board stand by the decision to retract the article.

